# Disinfection of Multidrug Resistant *Escherichia coli* by Solar-Photocatalysis using Fe-doped ZnO Nanoparticles

**DOI:** 10.1038/s41598-017-00173-0

**Published:** 2017-03-07

**Authors:** Sourav Das, Sayantan Sinha, Bhaskar Das, R. Jayabalan, Mrutyunjay Suar, Amrita Mishra, Ashok J. Tamhankar, Cecilia Stålsby Lundborg, Suraj K. Tripathy

**Affiliations:** 1 School of Biotechnology, Kalinga Institute of Industrial Technology (KIIT University), Bhubaneswar, 751024 India; 2 Department of Life Sciences, National Institute of Technology, Rourkela, 6150 India; 3 Department of Public Health Sciences, Karolinska Institutet, SE 17177 Stockholm, Sweden; 4 School of Applied Sciences, KIIT University, Bhubaneswar, 751024 India; 5 Center of Industrial Technology, KIIT University, Bhubaneswar, 751024 India

## Abstract

Spread of antibiotic resistant bacteria through water, is a threat to global public health. Here, we report Fe-doped ZnO nanoparticles (Fe/ZnO NPs) based solar-photocatalytic disinfection (PCD) of multidrug resistant *Escherichia coli* (MDR *E. coli*). Fe/ZnO NPs were synthesized by chemical precipitation technique, and when used as photocatalyst for disinfection, proved to be more effective (time for complete disinfection = 90 min) than ZnO (150 min) and TiO_2_ (180 min). Lipid peroxidation and potassium (K^+^) ion leakage studies indicated compromisation of bacterial cell membrane and electron microscopy and live-dead staining confirmed the detrimental effects on membrane integrity. Investigations indicated that H_2_O_2_ was the key species involved in solar-PCD of MDR *E. coli* by Fe/ZnO NPs. X-ray diffraction and atomic absorption spectroscopy studies showed that the Fe/ZnO NPs system remained stable during the photocatalytic process. The Fe/ZnO NPs based solar-PCD process proved successful in the disinfection of MDR *E. coli* in real water samples collected from river, pond and municipal tap. The Fe/ZnO NPs catalyst made from low cost materials and with high efficacy under solar light may have potential for real world applications, to help reduce the spread of resistant bacteria.

## Introduction

With supplies of fresh water diminishing and population increasing, water scarcity is set to become a major global problem. Considering population and water demand trends, it is predicted that about one third of the world’s population will be affected by illness and poverty due to water scarcity in 2025^1, 2^. The several dimensions of water scarcity, namely availability or difficulties in finding a reliable source of safe water, especially in arid regions, make the wastewater reuse an interesting option for augmenting available water supplies^3, 4^. Conventionally, wastewater is either treated using established treatment technologies and reused for secondary applications or discharged directly without any treatment into the water bodies^5^. Wastewater reuse offers some benefits like decrease in water scarcity pressure, and it becomes a contribution toward a more integrated management of urban water resources, but, if not planned, properly managed and implemented, it can have serious public health concern^6, 7^. One of the major risks arises from the presence of pathogenic microorganisms in wastewater and it is especially worrisome when the treated wastewater is contaminated with multidrug resistant (MDR) microorganisms^8–10^. Wastewater treatment plants are suspected to be one of the major anthropogenic sources for release of antibiotics, MDR bacteria and antibiotic resistant genes (ARG) into the environment^11^. In low income countries where hospital wastewater is treated along with municipal effluent or discharged without any treatment, the situation becomes more critical^12^. In particular, MDR bacteria, carrying antibiotic resistance genes that can contaminate the community water sources and can transfer their resistance to normal pathogens. This results in a decrease of antibiotic therapeutic potential against pathogens and, finally, may pose a severe threat to public health^13, 14^. Development of water disinfection technology to remove MDR bacteria is still a scientific and technical challenge since conventional methods such as chlorination and ozonation have shown disadvantages related to the formation of potentially hazardous disinfection by products (DBPs)^15, 16^. Additionally MDR bacteria are known to have genes which may repair DNA and allow them to regrow after the disinfection process^17^. Among alternative disinfection techniques proposed, heterogeneous photocatalysis has been successfully investigated for the removal of a wide range of contaminants^18–23^. Semiconductor nanoparticles (NPs), upon irradiation with light of proper wavelength (including sun light) are known to generate reactive oxygen species (ROS) such as hydroxyl radicals (·OH) which have been successfully employed for deactivation of pathogenic bacteria. Current semiconductor NPs based catalysts in vogue are mostly made up of costlier materials viz. TiO_2_ and usually are doped with Nobel metals (Ag or Pt). If suitable photocatalyst is designed with cheaper materials which will have higher disinfection efficiency at lower dose, then it will reduce the operational and capital cost and this process could become an attractive option for wastewater treatment, particularly in case of small communities and resource constraints settings^24^. To the best of our knowledge reports about the effect of photocatalysis on inactivation of MDR bacteria is relatively scarce. Tsai *et al.* reported the photocatalytic oxidation of antibiotic resistant *Staphylococcus aureus* and *Acinetobacter baumannii* by TiO_2_ NPs^25^. Xiong *et al.* showed the inactivation of antibiotic resistant *E. coli* (ATCC 700891) by UVA/LED/TiO_2_ system^26^. Similarly TiO_2_ assisted disinfection of resistant *E. coli* isolated from urban wastewater in presence UV and solar light was reported by Rizzo and coworkers^27^. Ferro *et al.* also investigated the solar driven advanced oxidation process for disinfection of resistant *E. coli* isolated from urban wastewater^28^. Most of the previous researches in this field have used TiO_2_ as the photocatalyst. In spite of these reports, practical exploitation of photocatalysis is limited. Hence, there is an urgent need to design alternative photocatalytic materials which will have higher disinfection efficiency at lower dose.

Here we report Fe/ZnO NPs assisted solar-photocatalytic disinfection (PCD) of MDR *E. coli* isolated from wastewater of a rural healthcare center in synthetic as well as natural water systems. Effect of process parameters on the disinfection efficiency was investigated and compared with that of commercial TiO_2_ (Degussa P25) and pure-ZnO. From a disinfection point of view, lipid peroxidation and potassium (K^+^) ion leakage studies were performed to verify compromisation of bacterial cell morphology. Effect of different reactive species on the disinfection efficiency was investigated to identify the molecular species responsible for disinfection of MDR *E. coli*. Field emission scanning electron microscopy (FE-SEM) and live-dead staining techniques were used to validate the PCD process.

## Results and Discussion

### Characterization of photocatalyst

#### Investigation of phase and crystal structure by X-ray diffraction (XRD) technique

**Figure 1:**
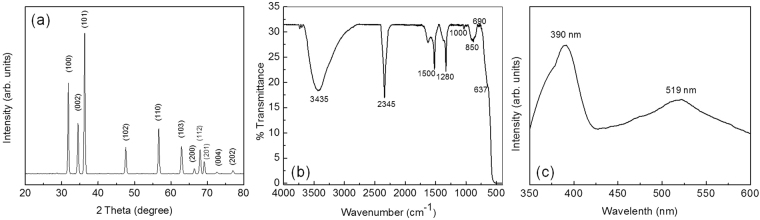
(**a**) XRD pattern, (**b**) FTIR spectrum, and (**c**) photoluminescence spectrum of Fe/ZnO NPs.

The XRD pattern for the Fe/ZnO and pure ZnO NPs are shown in Fig. 1(a) and Supplementary Figure S1(a) respectively. Undoped material has shown most of the characteristic peaks corresponding to ZnO wurtzite structure (JCPDS 36-1451) with P6_3_mc space group symmetry. The peaks at 2θ = 31.72°, 34.46°, 36.28°, 47.61°, 56.66°, 62.81°, 66.39°, 67.94°, 69.13°, 72.59° and 76.96° were identified as (100), (002), (101), (102), (110), (103), (200), (112), (201), (004) and (202) planes of wurtzite phase of ZnO (JCPDS 36-1451). Mean crystallite diameter (MCD) of the material is calculated using Scherrer’s equation and found to be 56 nm^24^. The XRD pattern of the Fe/ZnO NPs has not shown any peak which could be attributable to any of the iron oxides (viz. Fe_2_O_3_, Fe_3_O_4_, FeO, etc.) or Zn–Fe–O (viz., ZnFe_2_O_4_, Zn_x_Fe_3−x_O_4_, etc.) compounds. This could probably be attributed to the good dispersion of Fe^3+^ ions into the structure lattice of the ZnO or to the minute amount of Fe^3+^ dopant that was used in the synthesis protocol. However, the XRD patterns show broadening of the peaks which may suggest the formation of particles in the nanophase regime and/or may indicate that the Fe doping into the lattice of ZnO inhibited the crystallization of samples^29^. Additionally, peaks were slightly shifted towards lower angle. This suggests that dopants may have resulted in lattice disorder and associated stresses which have supported the growth of smaller particles^25^. This phenomenon can be explained by considering the difference in size of the ionic radii of Fe^3+^ (0.067 nm) and Zn^2+^ (0.083 nm) ions. The lattice parameters of Fe/ZnO and ZnO NPs were calculated from the XRD and were found to be; a = b = 3.246 Å, c = 5.2053 Å and a = b = 3.248 Å, c = 5.2058 Å, respectively. Lattice parameters of Fe/ZnO were slightly less than those of ZnO, suggesting that the Fe^3+^ ions were doped into the ZnO crystal lattice without changing the wurtzite structure^30^.

#### Investigation of surface groups by Fourier transform Infrared (FTIR) spectroscopy

The composition/functional property of Fe/ZnO NPs were analyzed with FTIR spectroscopy at room temperature in an acquired range of 500–4000 cm^−1^. Figure 1(b) shows the FTIR spectra of the calcined Fe/ZnO NPs. The broad band around 3400 cm^−1^ may correspond to O–H stretching mode of hydroxyl groups whereas the strong peak at 2345 cm^−1^ resembles to the stretching mode of acidic O–H group, which arises in the range of 2400–3300 cm^−1^. The small vibration appearing at 1630 cm^−1^ may belong to the stretching peak of C=O group^31^. Vibration peaks at 1500 and 1280 cm^−1^ corresponds to C–H bending and C–O stretching mode respectively^32, 33^. The peaks at 1630 and 637 cm^−1^ may correspond to Zn–O stretching and deformation vibration, respectively^34^.

#### Investigation of optical property by photoluminescence (PL) spectroscopy

Figure 1(c) and Supplementary Figure S1(b) shows the results of room-temperature PL spectra of Fe/ZnO and pure ZnO NPs respectively. ZnO NPs has two distinct peaks, one intense peak in UV-region (405 nm) and another broad peak in the visible region (518 nm). In case of ZnO, the UV-emission which is also known as near band edge emission (NBE) is expected to originate due to the recombination of the free excitons through an exciton-exciton collision process while visible emission known as deep level emission is attributed to the single ionized oxygen vacancy and may arise from the recombination of a photogenerated hole with the single ionized charged state of the defect in ZnO^35^. Egelhaaff *et al.* demonstrated that the defect level emission is originated due to the radiative transitions between shallow donors (related to oxygen vacancies) and deep acceptors (related to zinc vacancies)^36^. In case of Fe/ZnO NPs, the peak intensity of UV emission is increased, while the visible emission intensity is slightly decreased. Additionally, the peak due to UV emission is also slightly shifted towards the lower wavelength. Considering these observations, it is expected that the Fe/ZnO NPs have fewer oxygen vacancies than that of pure ZnO NPs^37^.

#### Investigation of material property by X-ray photoelectron spectroscopy (XPS)

Chemical environment of the pure and Fe-doped ZnO NPs are investigated by XPS and results are shown in Supplementary Figure S2 (supporting data). No remarkable change in the binding energy was noticed for Zn 2p_3/2_ and 2p_1/2_. However, a clear shift in the O 1 s position is observed after Fe doping. Therefore, it is expected that Fe doping in ZnO NPs may have decreased the oxygen deficiency, which may have resulted in the change of optical properties^38^. We have also tried to analyze the Fe 2p_3/2_, which is usually obtained ≈710 eV. However the spectrum contains large amount of noise and it was too difficult to identify any specific peak. This could be due to the fact that very small amount of iron is used for doping^39^.

#### Investigation of morphology by Transmission Electron Microscopy (TEM)

**Figure 2:**
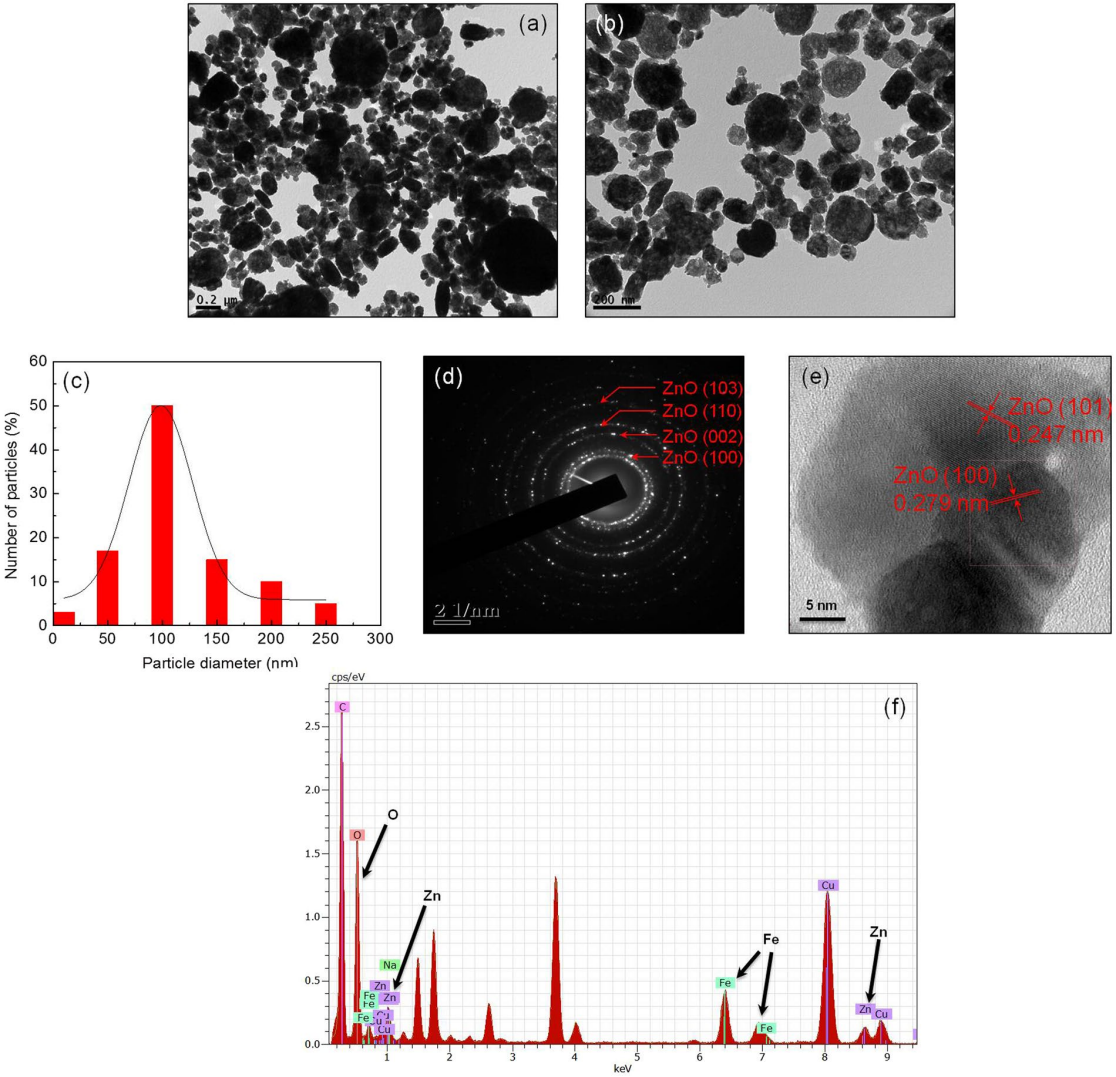
TEM images (**a**,**b**), size distribution graph (**c**), SAED (**d**), HRTEM image (**e**), and EDAX (**f)** pattern of Fe/ZnO NPs.

Morphology of the doped NPs is investigated by TEM analysis and results are shown in Fig. 2. Near spherical particles are observed [Fig. 2(a,b)]. Figure 2(c) shows the size distribution of the resultant particles. Most of the particles are in the range of 80 to 100 nm [Fig. 2(c)]. Comparative analysis of XRD and TEM results has suggested the formation of polycrystalline materials. Different TEM SAED pattern rings are indexed in Fig. 2(d). High resolution TEM (HRTEM) images are shown in Fig. 2(e) and the material is found to have inter-planer spacing of ≈0.247 nm which corresponds closely to the (101) plane of ZnO^37, 38^. TEM Energy Dispersive X-ray Spectrometry (EDS) technique is used to understand the chemical composition of the NPs. As shown in Fig. 2(f), Fe/ZnO NPs have shown clear peaks corresponding to Zn, O, and Fe. Peaks of Cu and C are also obtained. These peaks may have come from the carbon grid on which NPs are deposited for TEM studies^38, 39^.

#### Estimation of band gap of ZnO and Fe/ZnO NPs from UV-visible spectra

The absorbance spectrum for Fe/ZnO NPs is measured at room temperature and is compared with the spectrum of undoped ZnO NPs (Supplementary Figure S3 in supporting data). The undoped ZnO spectrum shows an absorption onset at approximately 410 nm, which is in good agreement with the intrinsic energy band gap of ZnO at 3.06 eV. The absorption spectra for all the Fe/ZnO is shifted to a longer wavelength (red shift) which was also indicated by the change in catalyst color from white to slight brownish appearance (with an band gap ≈2.91). The shift of absorption towards the red part of the spectra with Fe^3+^ doping could be attributed to sp–d exchange interactions between the band electrons in ZnO and the localized d electrons of the Fe^3+^ ^40^. However, absorbance shifting of Fe^3+^ doping samples to the visible region can be attributed to Burstein–Moss effect, which the level of Fermi energy combines to the conduction band because carrier concentration of doping increased as reported earlier by Nair *et al.* ^41^. Fe^3+^ ions in the valance band works as defects sits to reduce band gap has been reported and within absorbance of the light, electron–hole pair was formed. Therefore, the energy needs to transition from valance to conduction was lower energy than band gap of undoped ZnO^41^. This increase absorbance in the visible light spectrum caused by iron doping is expected to enhance the photocatalytic activity of Fe/ZnO NPs^42^.

#### Solar-PCD of MDR *E. coli* by Fe/ZnO NPs

**Figure 3:**
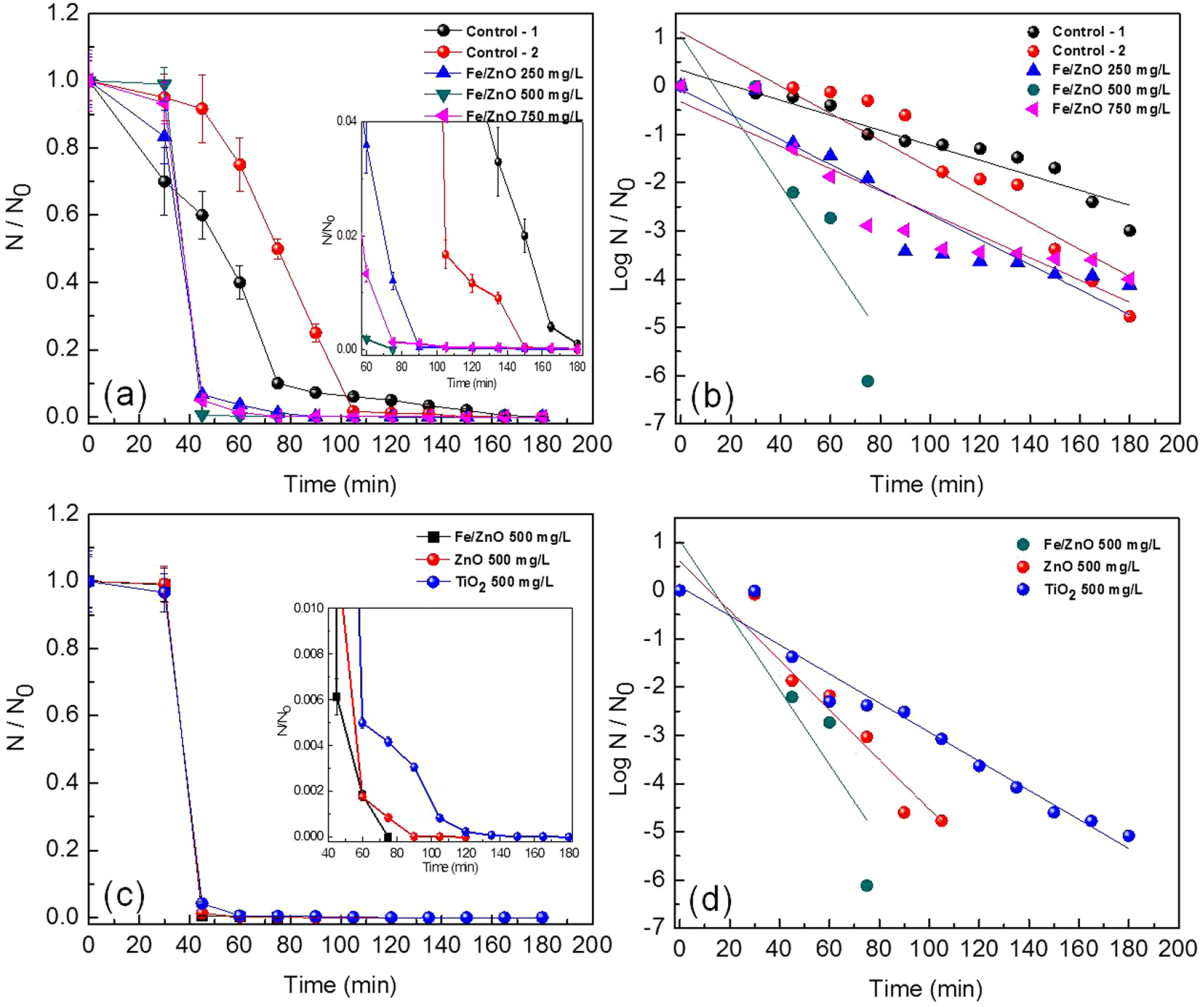
(**a**) Effect of Fe/ZnO NPs loading on the solar-PCD kinetics of *MDR E. coli*, (**b**) Linear fitting plots of PCD kinetics according to Chick-Watson model, (**c**) Effect of different catalysts on the solar-PCD kinetics of MDR *E. coli* at a catalyst loading of 500 mg/L, (**d**) Linear fitting plots of PCD kinetics of different catalysts according to Chick-Watson model at a catalyst loading of 500 mg/L. Initial MDR *E. coli* concentration = 1.2 × 10^7^ CFU/mL, Temperature = 35 ± 2 °C, pH = 6.5. Error bars indicate the standard deviation of replicates (n = 3).

The time dependent disinfection of MDR *E. coli* as a function of Fe/ZnO catalyst concentrations is shown in Fig. 3(a). It is observed that the disinfection efficiency increased with an increase in the catalyst loading up to the optimum point (500 mg/L) i.e. 6.1141 log reductions in 75 min. The disinfection rate decreased with further increase in the catalyst dosage to 750 mg/L i.e. 4 log reductions in 180 min. In the present report, efficiency at 500 mg/L of NP loading was higher than that of other concentrations, as evident from the faster decrease in cell viability [Fig. 3(b)]. When compared with the two control experiments i.e Control-1 (Dark control) and Control-2 (light control) where the disinfection rate was 3 log reductions in 180 minutes and 4.7781 log reductions respectively, PCD was found to be more efficient. Thus, 500 mg/L catalyst concentration was found optimum for efficient disinfection of MDR *E. coli*. With a lower dosage than 500 mg/L, it is expected that there is an insufficient usage of the incident solar light by the catalyst resulting in lesser generation of ROS/oxidizing species to be used for photocatalytic process. With an increasing dosage of catalyst above 500 mg/L, chances of more ROS generation persists but due to simultaneous increase in the turbidity of the slurry solution and possible light shielding effect of the catalyst, utilization of the incident photons decline resulting in decreasing efficiency of disinfection. In Control-1 due to absence of light, the semiconductor catalyst fails to generate ROS and in Control-2 in presence of sunlight and no catalyst, substantial disinfection upto 5 log reductions was observed in 180 minutes, which possibly may be because exposure to sunlight transforms a viable and culturable bacteria to a viable but non-culturable (VBNC) state^43^ that finds difficulty in growing in nutrient medium when plated and reactivates within 24 h post disinfection as will be discussed later. As shown in Figure S4 (supporting data), under the optimized reaction condition complete disinfection of normal *E. coli* was achieved in a lesser time (75 min) than that of its MDR variant (90 min). Rizzo *et al.* has also observed that antibiotic resistance strains has slower inactivation kinetics with conventional techniques (e.g. chlorination and UV-irradiation)^27^.

Reaction temperature is expected to have a significant impact on the rate of disinfection process. In the present case the operational temperature was maintained at 35 ± 2 °C. As shown in Figure S5 (supporting data), the rate of PCD increased with increase in the reaction temperature. A remarkable increase in the disinfection rate was not observed. At 65 °C, the highest rate of bacterial disinfection was noticed which may be attributed to accelerated cell death at higher temperature (particularly in the initial 30 min of the reaction)^44^.

The PCD efficiency of Fe/ZnO was compared with commonly used photocatalysts i.e. ZnO and TiO_2_, and as shown in Fig. 3(c,d) it was observed that ZnO results in complete disinfection of MDR *E. coli* in nearly 150 min but for TiO_2_, 5.089 log reductions occurred in nearly 180 min. However, Fe/ZnO NPs have shown a complete disinfection in 90 min (≈7 log reduction), thus suggesting the better efficiency of proposed nanophotocatalyst. It is expected that, the localized electronic states of the Fe served as trapper of photo-generated charge carrier under solar irradiation further delaying the electron hole recombination and increasing the efficiency of PCD unlike normal ZnO and TiO_2_. As per the XRD results, it can be inferred that Fe ions doped in ZnO NPs reduced its average crystallite size and simultaneously narrowed the band gap (as evident from UV-visible spectra) and this may be one of the important cause for increasing the PCD efficiency^45^. Moreover under solar irradiation electron from Fe^3+^ (in Fe/ZnO semiconductor photocatalyst) can be photoexcited to conduction band (CB) leaving behind Fe^4+^ and holes in the valence band (VB). The electron shuttled in the conduction band could be transferred to the oxygen to produce superoxide radical (i.e. ·O_2_^−^), whereas Fe^4+^ and holes in the valence band could react with a surface hydroxyl group to produce a hydroxyl radical (OH·). These reactive species along with some other ROS may have initiated the photocatalytic disinfection reaction. This further may lead to more efficient disinfection of MDR *E. coli* by Fe/ZnO in comparison to normal ZnO and TiO_2_.

#### Investigation of the role of different ROS on solar-PCD of MDR *E. coli* by Fe/ZnO NPs

**Figure 4:**
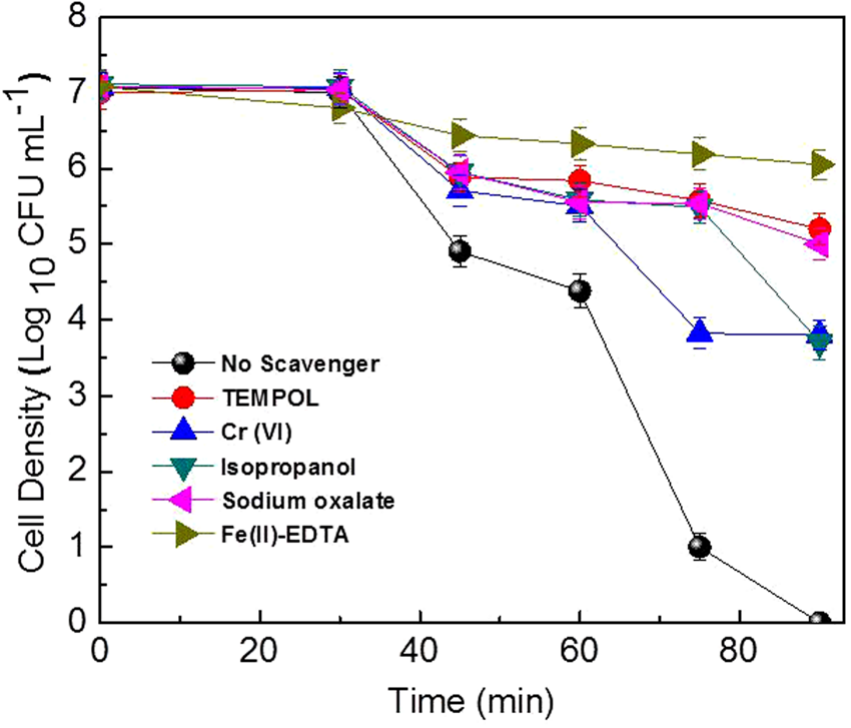
Effect of different scavengers on the solar-PCD of *MDR E. coli* in presence of Fe/ZnO NPs. Initial MDR *E. coli* concentration = 1.2 × 10^7^ CFU/mL, Temperature = 35 ± 2 °C, pH = 6.5, [Fe/ZnO NPs] = 500 mg/L. Error bars indicate the standard deviation of replicates (n = 3).

It has been suggested that in presence of photocatalyst and sunlight various ROS may be generated, which play a significant role in killing the pathogens. Thus, it is very important to investigate the effect of individual ROS in this disinfection process. Different ROS scavengers were used to inhibit each of these components specifically, to discriminate each of their contributions to the PCD (Fig. 4). Isopropanol (0.5 mM) was used to scavenge ·OH; Sodium oxalate Na_2_C_2_O_4_ (0.5 mM) was used to scavenge VB h^+^; TEMPOL (2 μM) scavenges the ·O_2_^−^; K_2_Cr_2_O_7_ (50 μM) quenches the CB e−^46^ and Fe(II)-EDTA salt (0.1 mM) was used for scavenging H_2_O_2_. In absence of any of these scavengers, 1.2 × 10^7^ CFU/mL of MDR *E. coli* could be completely disinfected within 90 min. When isopropanol and K_2_Cr_2_O_7_ was added, ≈55% decrease in bacterial cell was noticed after 90 min (≈3.0 log reduction), indicating involvement of ·OH and e− in this photocatalytic disinfection to a lesser extent. However, the addition of sodium oxalate and TEMPOL inhibits MDR *E. coli* disinfection upto ≈75% in 90 min. Only ≈2 log reductions in each of these cases was observed, indicating a substantial contribution of h^+^ and ·O_2_^−^ in the disinfection process. As mentioned in literature, not only these four reactive species are generated during the PCD process, but also other reactive species with better oxidation efficiency like H_2_O_2_ are generated within the system^47^. When a H_2_O_2_ scavenger in the form of Fe(II)-EDTA was added to the present system, only 1 log reduction of MDR *E. coli* was observed thus confirming nearly 86% decline in the disinfection efficiency in comparison to no scavenger data. Maness *et al.* have previously reported about the generation of H_2_O_2_ in the system during PCD^48^. Doping with transition metal has advantages with respect to increase in the photocatalytic property of the semiconductor photocatalyst under visible light irradiation by decreasing the band gap or on the other hand to delay the recombination time of the electron and hole generated in the CB and VB respectively. The presence of oxygen in the system delays the recombination of electron hole pair, while letting the formation of superoxides radical (·O_2_^−^). This ·O_2_^−^ radical forms the hydroperoxyl radical (HO_2_·) on protonation and finally H_2_O_2_ with further protonation^15^. When the HO_2_· concentration in the system increases, it is proposed that they combine to generate more H_2_O_2_ as evident from the present results^46^. There has been evidence that H_2_O_2_ can also be produced in the valence band by coupling of two ·OH in the bulk solution. In addition to generation of H_2_O_2_, co-existence of HO_2_· and ·O_2_^−^ radical in the photocatalytic reaction system can prolong the recombination time of the electron hole pair due to their electron scavenging nature. Thus it is proposed that H_2_O_2_ is the key ROS involved in solar-PCD of MDR *E. coli* by Fe/ZnO NPs. In case of TiO_2_ and ZnO it was found that the most reactive species involved in PCD is ·OH radical, though a good influence of H_2_O_2_ is also reported^49–52^.

#### Investigation of bacterial membrane damage by lipid peroxidation

**Figure 5:**
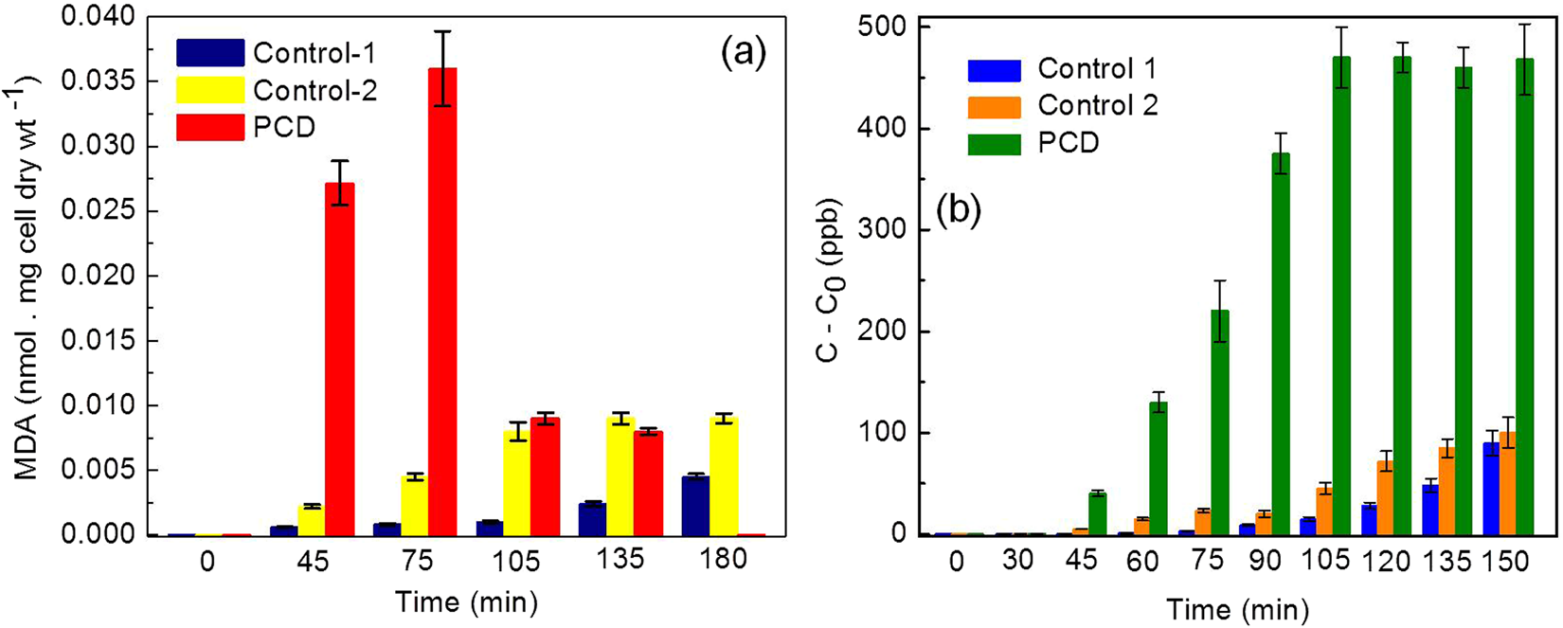
(**a)** Lipid peroxidation kinetics of MDR *E. coli* induced by Fe/ZnO NPs and (**b**) leakage of K^+^ ion from *MDR E. coli* cells subjected to PCD in presence of Fe/ZnO NPs. Initial *MDR E. coli* concentration = 1.2 × 10^7^ CFU/mL, Temperature = 35 ± 2 °C, pH = 6.5, [Fe/ZnO NPs] = 500 mg/L. Error bars indicate the standard deviation of replicates (n = 3).

It is well known that bacterial membranes are the primary target in PCD. The ROS generated in the system leads to peroxidation of the membrane lipids releasing malondialdehyde (MDA) which is said to be the key biomarker of membrane lipid peroxidation^48^. So biochemical tests were performed to quantify the time dependent release of MDA in photocatalytic treatment. It was found that neither Fe/ZnO at concentration 500 mg/L in dark condition (Control-1) nor sunlight alone without Fe/ZnO (Control-2) for 180 min has lead to substantial lipid peroxidation as shown in Fig. 5(a). Small amount of MDA that has been released in the system may be because of the action of the catalyst under dark condition. Whereas impact of solar-disinfection in Control-2 may lead to generation of minute amount of hydroxyl radicals in the reaction system which in turn can peroxidize the membrane lipids. In contrast, during the PCD with 500 mg/L of Fe/ZnO within 75 min exposure, an exponential increase in MDA production i.e. upto 0.036 nmol/mg cell dry weight was observed as a result of lipid peroxidation. The result obtained is approximately 8 times and 4 times greater than the Control-1 and Control-2 respectively. There is a direct correlation between the number of bacteria disinfected and generation of MDA. Gram-negative bacteria are known to have phospholipid membranes with repeated arrangement of lipids. They are mainly composed of glycerol-phospholipids, like phosphatidylethanolamine, phosphatidylglycerol, and cardiolipin^53^. The repeated arrangement allows the initiation of a radical chain reaction in presence of ROS. It has already been established through the ROS scavenger experiment that H_2_O_2_, ·O_2_^−^ and h^+^ are the major contributors in this case to initiate the photocatalytic membrane damage process. In presence of Fe/ZnO NPs the reduced Fe ion (Fe^2+^) may combine with H_2_O_2_ to generate hydroxyl radical via fenton process. Superoxide radical can also react with the oxidized Fe ion (Fe^3+^) to form Fe^2+^ through Haber-Weiss reaction and in turn could affect the redox cycling^54^. The former reactive species (·OH) may attack unsaturated membrane lipids to yield lipid radical. This in the presence of oxygen it is expected to yield a lipid peroxyl radical which is capable of abstracting hydrogen from the adjacent unsaturated lipid to give lipid hydroperoxide and a lipid radical again. Thus the chain of reaction continues until all the membrane unsaturated lipids are converted into lipid hydroperoxides and subsequent production of malondialdehyde (a stable by-product of membrane lipid peroxidation). Thus the amount of MDA generated is directly correlated to the H_2_O_2_ generated in the system^53, 54^. An interesting trend i.e. the decrease in MDA concentration after 75 min was observed in photocatalytic treatment as similar to the results obtained by Maness *et al.* ^48^. The possible justification for this might be that, after 75 minutes the active sites of the catalyst are vacant as there is no more organic target left in the reaction system in the form of bacteria as confirmed from the CFU count data in Fig. 3(a). During this time the MDA being an organic compound is expected to be the prime target of the photocatalyst and might suffer photocatalytic mineralization^48^. This process is considered beneficial because MDA generated in the water is a carcinogen and its mineralization is acceptable as far as real water treatment applications are concerned.

#### Investigation of bacterial membrane damage by analysis of K^+^ ion leakage

The leakage of K^+^ was studied to determine the alteration in cell permeability as a consequence of membrane damage during PCD and results are shown in Fig. 5(b). It was observed that under photocatalytic treatment, MDR *E. coli* shows an exponential increase in K^+^ leakage starting from 0–470 ppb in 105 min. These results correspond to the membrane damage analysis through lipid peroxidation assay^48^. The K^+^ ions are known to participate in regulating the polysome content and protein synthesis of the bacterial cell. As a consequence of ROS generated in the system the outer cell membrane undergoes lipid peroxidation and dys-functioning with a subsequent release of K^+^ ion^55, 56^. This may further lead to loss in bacterial cell viability. In contrast, the two controls, Control-1 and Control-2 with the presence of Fe/ZnO in the dark and absence of Fe/ZnO in light, the membrane damage was not remarkable. The maximum K^+^ leakage in both this case was up-to 90 and 100 ppb respectively. The minor increase of K^+^ in the control resulted from a portion of cell undergoing natural death, or damage caused by radicals generated as a result of Fenton process and normal solar light exposure.

#### Investigation of bacterial membrane damage by LIVE/DEAD staining

**Figure 6:**
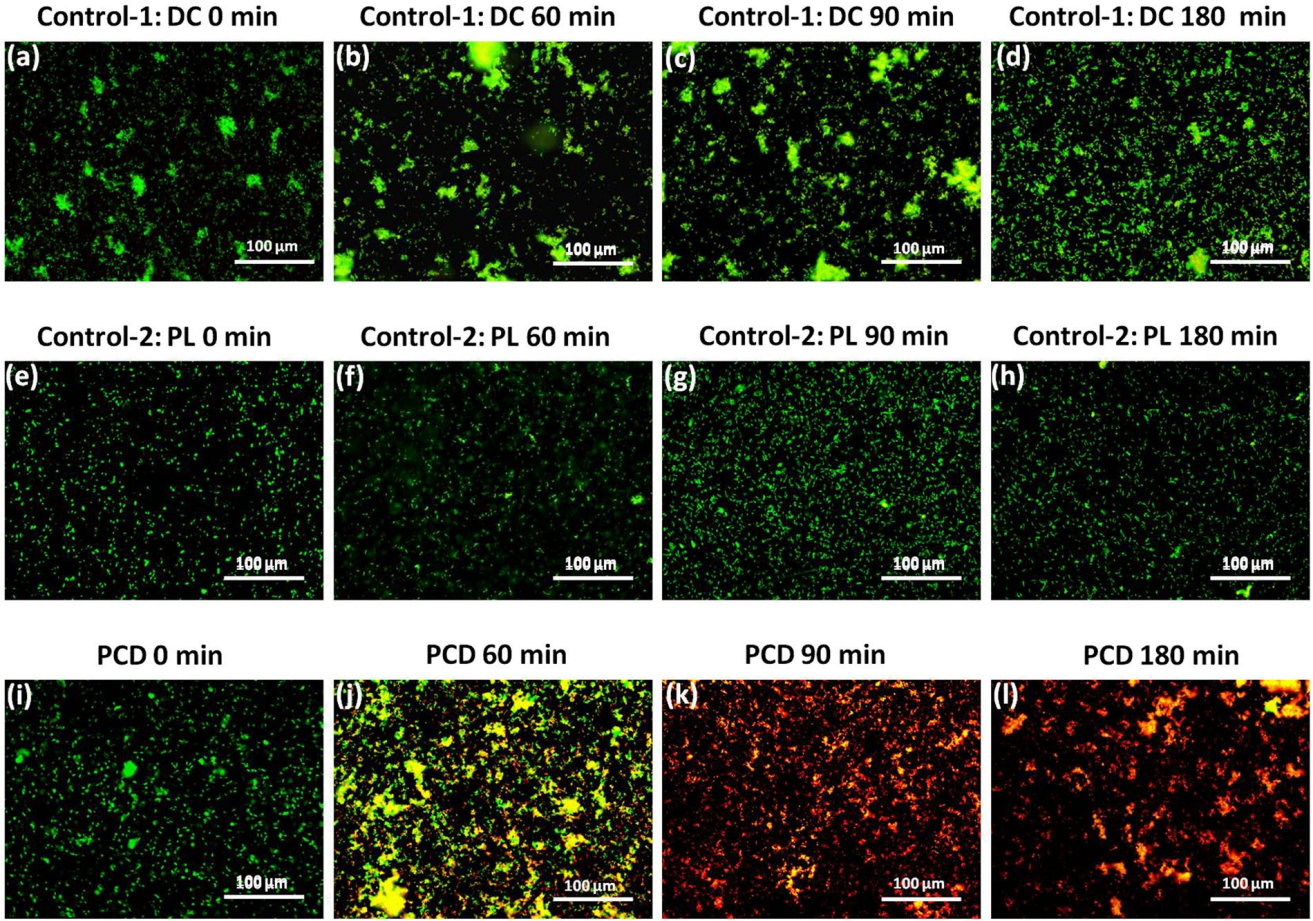
(**a**–**d**) Shows the fluorescent microscopic images dark control (control-1) treatment of MDR *E. coli* without the presence of light at 0, 60, 90 and 180 min respectively (with [Fe/ZnO NPs] = 500 mg/L). (**e**–**h**) Shows fluorescent microscopic images the light control (control-2) treatment of *MDR E. coli* without the presence of catalyst at 0, 60, 90 and 180 min respectively. (**i**–**l**) Shows the fluorescent microscopic images of *MDR E. coli* that was subjected to solar-PCD in presence of Fe/ZnO NPs ([Fe/ZnO NPs] = 500 mg/L) at 0, 60, 90 and 180 min treatment respectively.

It has been suggested earlier through the plate count method that in case of photocatalysis, in comparison to the two control experiments the extent and rate of disinfection was much higher. To further confirm the effect of Fe/ZnO NPs assisted PCD on the structural integrity of the bacteria cell membrane, the fluorescence staining assays were performed on photocatalytically treated MDR *E. coli* along with the two controls (dark control and light control). As shown in Fig. 6(a–d), the dark control experiment revealed that Fe/ZnO NPs in absence of light have very less detrimental effect on the membrane integrity of the bacteria. The green fluorescence with very less yellowish and red fluorescence (hallmark of damaged membrane) still persists after 180 min. Similar kind of observation was found on the light irradiated bacteria without catalyst where there was no remarkable decrease in green fluorescence of bacteria after 180 min, thus confirming the well maintained integrity of bacterial membrane after photolytic treatment [Fig. 6(e–h)]. However, in the presence of Fe/ZnO NPs, after 60 min irradiation by sun-light, ≈50% of the cells showed red or pale yellow fluorescence indicating bacteria on the verge of losing their membrane integrity [Fig. 6(j)]. After 90 and 180 min irradiation, hardly any living bacteria can be observed as the green fluorescence is completely lost and almost all the bacteria are stained red and pale yellow. This observation confirms complete PCD of the target bacteria [Fig. 6(k,l)]. These data corresponds well to the plate count data obtained for PCD process. Due to continuous generation of ROS over the catalyst surface, the bacterial cell membrane is expected to be the prime target^48^, suggesting that the fluorescent LIVE/DEAD staining technique is a straight and precise method to get confirmatory results^49^.

#### Investigation of bacterial membrane damage by Field Emission Scanning Electron Microscopy (FESEM) analysis

**Figure 7:**
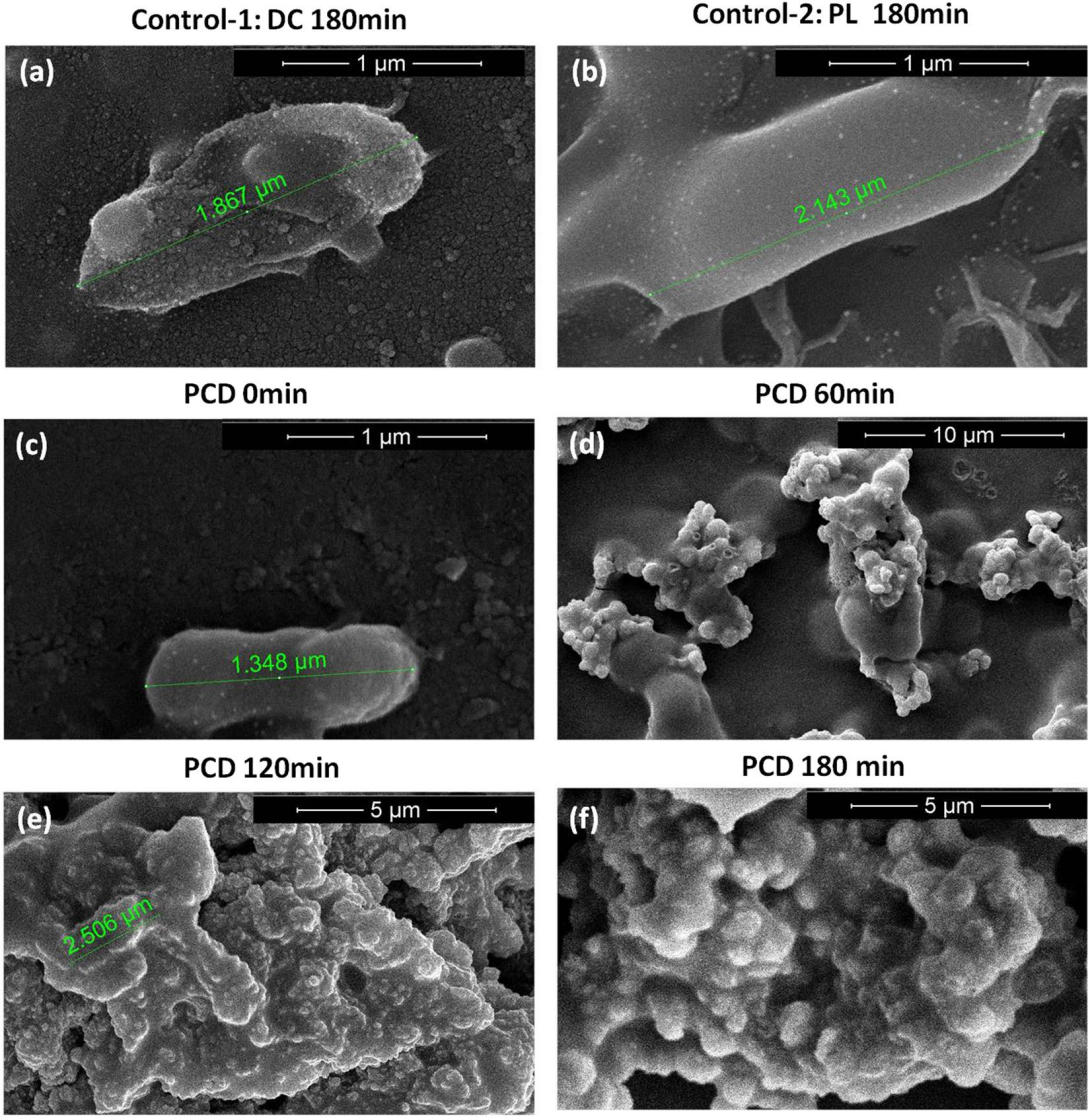
(**a**,**b**) Shows the FESEM images dark control (control-1) (with [Fe/ZnO NPs] = 500 mg/L) and photolysis treatment (control-2) of MDR *E. coli* at 180 min respectively. (**c**–**f**) Shows the FESEM images of MDR *E. coli* that was subjected to solar-PCD in presence of Fe/ZnO NPs ([Fe/ZnO NPs] = 500 mg/L) at 0, 60, 90 and 180 min treatment respectively.

To better understand the progress in disinfection of the target bacteria during photocatalysis, the morphology of MDR *E. coli* with respect to change in membrane structure and integrity were observed using FESEM. In the Fig. 7(a) dark control shows photocatalyst adsorbed on the bacteria surface thus giving a rough texture to the surface of bacteria. However the bacteria were found to be intact with less morphology alteration. In light control [Fig. 7(b)] no damage to the bacteria is visible and the effect of ROS produced was found to be very less detrimental. As shown in Fig. 7(c), the untreated MDR *E. coli* exhibited even interiors and intact structure within the size range of 1~1.5 µm along the anterior posterior axis. Figure 7(d–f) shows that within 60 min of photocatalytic treatment blebs in the bacterial membrane could be observed which intensified with time, showing completely perturbed membrane morphology and loss in intracellular components of the cell with 90 and 180 min of PCD process. These damages could be because of the generation of ROS like H_2_O_2_ and ·O_2_^−^ in the system which were found to be the major reactive species involved in this study. The result corresponds well with the K^+^ ions leakage study. The potassium leakage occurs as a result of disrupted or perturbed membrane integrity and according to the results obtained earlier in Fig. 5(b) after 60 min, a significant amount of K^+^ ion have leaked till 120 min. In contrast the two control experiments did not infer much cellular damage even after 180 min. The disrupted bacteria in photocatalytic treatment were the consequence of membrane peroxidation and oxidative stress unlike the two controls where the ROS production was less. Hence this result corresponds well with the Live/dead staining data thus confirming the permanent detrimental effect of photocatalysis over pathogenic MDR bacteria^57^.

#### Reactivation study of bacteria after PCD

**Figure 8:**
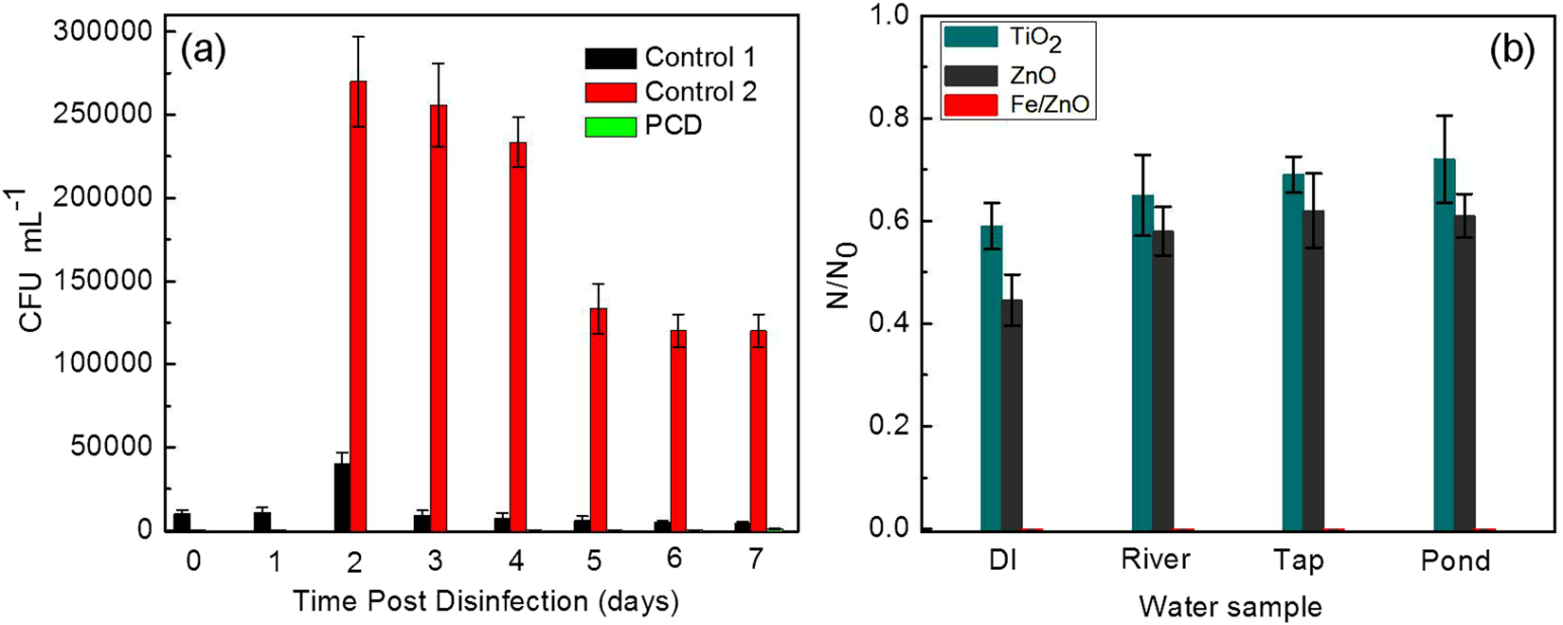
(**a**) Post-disinfection reactivation efficiency of MDR *E. coli* and (**b**) Effect of different photocatalysts on the relative reduction in the MDR *E. coli* cell count (N/N_0_) in real water samples after 180 min of solar irradiation at a catalyst loading of 500 mg/L.

The reactivation efficiency of MDR *E. coli* was studied up-to 7 days post PCD and the results are shown in Fig. 8(a). The MDR *E. coli* bacteria treated with photocatalysis shows very less reactivation in 7 days i.e. from 10^2^~10^3^ CFU mL^−1^ from 4^th^ day onwards till 7^th^ day which is not that substantial in comparison to the two control experiments. The probable reason behind the lesser reactivation of MDR *E. coli* after photocatalysis might be the long lasting damage effect due to substantial amount of H_2_O_2_ generation in the system. This along with the Fe^3+^ ion of doped ZnO in the slurry is expected to generate hydroxyl and hydroxyperoxyl radical (Fenton or Photo-Fenton effect) and hence may hinder the damage repair mechanism of the target pathogen^58^. According to previous reports, trace amounts of “free” iron in the system through Fenton/Habber–Weiss reaction cycle can produce hydroxyl radicals and hence can hinder the survival ability of bacteria^54^. In the Control-1 (Dark control) a very unusual trend of reactivation was observed. In the first two days the number of bacteria colonies increase up-to 4 × 10^4^ CFU/mL followed by a decreasing CFU count to 4.5 × 10^3^ CFU/mL on the 7^th^ day. The possible explanation for this observation could be either due to toxic effect of Fe/ZnO NPs on prolonged exposure or depletion in nutrient and carbon source within the reaction system which slows down the rate of multiplication. A similar kind of trend was observed in the Control-2 (Light control experiment), where in the first two days reactivation in the number of colonies up-to 2.7 × 10^5^ CFU mL^−1^ was observed which till the 7^th^ day, slumps down to 1.2 × 10^5^ CFU mL^−1^. The probable explanation for this increase might be that some bacteria within the reaction system survived the disinfection process and activated the damage repair mechanisms under dark conditions, thus leading to further multiplication. Continuous mechanical stress through the means of stirring in absence of catalyst for the next 5 days with simultaneous unavailability of sufficient nutrients in the system did not allow the bacteria to reactivate further to a greater extent^59, 60^. Presence of catalyst in the system; say in photocatalysis and dark control experiment favors the catalyst microbe interaction and thus may decrease the impact of mechanical stress on the target pathogen^58^.

#### Stability of the catalyst after PCD

Chemical species present in the reaction slurry may change the crystal structure of the photocatalyst and may reduce the photocatalytic activity. It is considered as one of the greatest challenges for practical application of PCD. The stability of the catalyst in post reaction condition was investigated using XRD and no alteration in crystal structure of Fe/ZnO NPs was observed suggesting its structural stability throughout the process (Figure S6 in supporting data). The intensity of the XRD peaks may have decreased because some amount of sample is lost during washing. It is known that leaching of metal ions could re-toxify the system and it could also be argued that Fe^3+^ and Zn^2+^ ions which are reported to show antimicrobial property may leach out of the system and hence, may be the actual cause of disinfection. To further eliminate this possibility, we have analyzed the water sample subjected to PCD by atomic absorption spectroscopy (AAS, Agilent Technologies Inc.) to detect the presence of metal ions. AAS analysis was done with treated water sample in triplicate and detectable amount of Fe^3+^ and Zn^2+^ ions were not noticed in any of the tests.

#### PCD efficiency in real water systems

**Figure 9:**
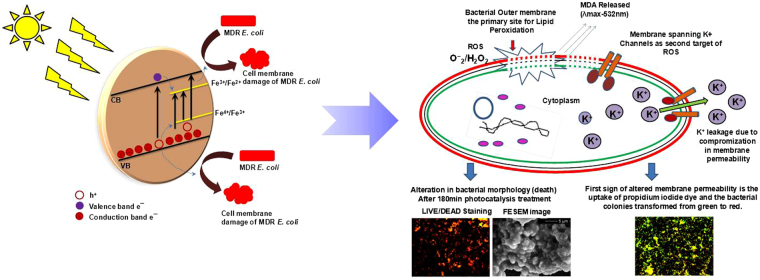
Schematic view of solar-photocatalytic disinfection of MDR *E. coli* in presence of Fe/ZnO NPs.

To evaluate applicability of the proposed disinfection technique i.e. Fe/ZnO NPs assisted solar-PCD in natural systems, when sterilized water samples from municipal tap, river and pond spiked with measured amount of MDR *E. coli* ^44^ were subjected to photocatalysis in presence of three different photocatalysts (Fe/ZnO NPs, ZnO and TiO_2_) maintaining a concentration of 500 mg/L, it was observed that [Fig. 8(b)], Fe/ZnO NPs exhibited superior disinfection profile as compared to pure ZnO and TiO_2_ in case of all the real water samples and the disinfection efficiency for natural waters as well as de-ionised water was similar. The various results obtained during the current experimentation suggest that Fe/ZnO NPs based photocatalytic system can be successfully used for disinfection of waterborne bacteria under solar irradiation. A schematic view of solar-photocatalytic disinfection of MDR *E. coli* in presence of Fe/ZnO NPs is shown in Fig. 9. Our laboratory has taken up further research to evaluate the versatility of the Fe/ZnO NPs based photocatalytic system.

## Conclusion

When a MDR *E. coli* strain was subjected to Fe/ZnO NPs mediated photocatalytic disinfection under solar irradiation, more than 99.9% disinfection of the targeted pathogen was achieved within 90 min. The disinfection profile was validated using Chick-Watson disinfection model. Our investigations suggested that H_2_O_2_ is the key ROS involved in solar-PCD of MDR *E. coli* by Fe/ZnO NPs. Quantitative analyses of K^+^ ion release and MDA assay proposed the damage of bacterial cell membrane to be by ROS generated during PCD. Study of effect of PCD on the morphology and structural integrity of the pathogen by fluorescence microscopy and FESEM analysis suggested that the targeted pathogen could not re-grow by using its DNA-repair mechanism. Disinfection achieved using the Fe/ZnO NPs was also validated for safety and also tested using real world water samples from municipal tap, pond and river. The developed photocatalytic process may be useful in designing an efficient and low cost water decontamination system.

## Materials and Methods

### Synthesis and characterization of Fe/ZnO NPs

In a 1000 mL conical flask 500 mL of 50 mM Zinc nitrate hexahydrate solution supplemented with equivalent concentration of stabilizing agent Trisodium citrate and 3 mol wt % Ferric chloride was prepared and stirred until a pale yellow transparent solution was obtained. The solution was heated at 80 °C for 15 min with constant stirring at 600 rpm followed by drop wise addition of 250 mL of aqueous NaOH solution using a burette. The solution was kept undisturbed for 2 h at 80 °C until the pale yellow solution transformed into opaque yellowish milky slurry. After saturation, the slurry was cooled and centrifuged at 12000 rpm for 15 min followed by repeated washing with distilled water, vigorous mixing and subsequent centrifugation to get rid of extra NaOH. After washing, the final precipitate were collected in a crucible and kept overnight in a hot air oven at 60 °C followed by sintering at 600 °C for 1 h in a muffle furnace to remove the organic contaminants. ZnO NPs were prepared in a similar manner except being supplemented with Ferric chloride in the initial solution. The morphology of the NPs was investigated by TEM (JEOL-JEM-2010) and FESEM. The crystal phase of all the materials was investigated by XRD (D/Max 2005, Rigaku). The surface modifications were studied using FTIR spectroscopy (Shimadzu 8201PC, Japan). All chemicals sourced from Merck.

### Preparation of bacterial cultures and evaluation of antibiotic resistance

Chikiti Public Health Centre in Berhamapur District of Odisha was selected as the site for hospital waste water sample collection because of its well equipped microbiology laboratory facility. MDR *E. coli* was obtained from the waste water of the above mentioned site. Antibiotic susceptibility test was done using Kirby-Bauer Disc diffusion test. The disc strength of different antibiotics was selected as per the Clinical and Laboratory Standards Institute (CLSI) guidelines. The choice of antibiotics was based on the CLSI guidelines and use of antibiotics in the study area for the treatment of infections caused by gram negative coliforms^59^. The diameter of inhibition zones was measured in millimeter thrice and the average value was taken. The strain was found resistant to 10 antibiotics (from four different groups namely, penicillin, cephalosporins, fluoroquinolones and tetracyclines (Table-1 supporting data) and thus was used for PCD experiments in presence of sun light using Fe/ZnO NPs. The strain was grown in a Luria-Bertani (LB) broth (SRL, India) at 37 °C in a shaking incubator (Labtech, India) at 180 rpm. At optical density (OD_600_)-0.8 corresponding to 10^8^ CFU/mL, the bacteria was harvested by centrifugation at 5000 rpm for 10 min followed by two rounds of washing with 0.9% normal saline solution (NSS) to get rid of extracellular bio-molecules from the target bacteria. All the glasswares and plasticwares were sterilized by autoclaving at 121 °C and 15 Psi for 20 min before being used for the study^60, 61^.

### Sun-light assisted PCD

PCD reactions were carried out in 500 mL glass reactor under continuous and controlled agitation (200 rpm). The reactor was kept in a vessel with a water circulating unit to maintain the reaction temperature. The temperature of the system was monitored by a digital thermometer and was maintained at 35 ± 2 °C during the experiment. MDR *E. coli* with a final cell density of 1.2 × 10^7^ CFU/mL were put in 300 mL of normal saline solution and reactions were performed with varying concentrations of Fe/ZnO NPs ranging from 250 to 750 mg/L. The set up was initially kept in dark for 30 min to attain equilibrium followed by exposure to solar light irradiation for 150 min (with an illuminance ≈100,000 ± 5000 lux). Samples were collected at every 15 min intervals. To check the inactivation of bacteria, 100 μL of the collected samples were serially diluted in sterile 0.9% NSS and 100 μL from the appropriate dilution was spread on LB agar plates. The plates were incubated overnight at 37 °C followed by viable cell count to obtain the rate of disinfection^44^. The reactor system post solar PCD experiment was kept under dark condition and continuous stirring to check the reactivation of bacteria. The reactivation assessment was done till 7 days of post PCD time with sampling and plating every 24 h. A comparative study of PCD using ZnO and commercial catalyst TiO_2_ (Degussa P25) with the optimum catalyst concentration of disinfection as obtained for Fe/ZnO was done to check the efficiency of the synthesized photocatalyst. Two control experiments were performed. 1) Under photolytic condition the bacterial population was exposed to solar irradiation in absence of Fe/ZnO, 2) Under non-photolytic condition, bacterial population was exposed to Fe/ZnO in the absence of light.

### Empirical disinfection kinetics

Empirical modeling was employed to understand the nature of PCD process. The present disinfection profile for *E. coli* is expected to be a pseudo-first-order reaction kinetics as proposed by the classical disinfection model of Chick and Watson^62, 63^ as represented below:

1 $$ln\frac{N}{{N}_{0}}=\,-K{[\chi ]}^{n}t$$

where, N_0_ and N are the initial and subsequent bacterial concentrations, respectively at the beginning of the process and after time ‘*t*’ respectively. ‘*K*’ is the disinfection kinetic constant, ‘*χ*’ is the concentration of disinfecting agent (photocatalyst in this case) and *‘n’* is the order of reaction.

### Determination of Lipid peroxidation

Malondialdehyde (MDA) is one of the key products of bacterial membrane peroxidation. Therefore estimation of MDA was performed by Lipid peroxidation Assay Kit (Sigma-Aldrich) according to the manufacturer’s protocol^48^. The samples were analyzed at 532 nm using UV-visible spectrophotometer (Shimadzu UV-1800). The concentration of MDA in the system was expressed in nanomoles of MDA released per mg dry weight of bacteria.

### Potassium ion (K^+^) leakage studies

To study the K^+^ leakage from photocatalytically inactivated bacteria, 2 mL sample (subsequent replenishment of an equal volume of 0.9% NSS was done to maintain reaction volume and integrity) was collected at regular time intervals (every 15 min) from the reaction system and was subjected to centrifugation^55^. The supernatant was analyzed using microwave plasma atomic emission spectrometer (4200, Agilent).

### Measurement of ROS

Various reactive species which are formed *in situ* as a result of semiconductor photocatalysis are H_2_O_2_, ·O_2_^−^, ·OH, h^+^, and e^−^^16^. To determine which one of the mentioned reactive species contributes towards Fe/ZnO mediated solar PCD of bacteria in this system, different individual scavengers were used to remove the respective reactive species. Various scavengers used in this study include Na_2_C_2_O_4_ for h^+^ (0.5 mM), Cr(VI) for e^−^(50 µM), EDTA-Fe(II) for H_2_O_2_ (100 µM), 2-propanol for ·OH (0.5 mM), and 4-hydroxy-2,2,6,6-tetramethylpiperidinyloxy (TEMPOL) for ·O_2_ (2 mM)^57^.

### Investigating the cell membrane damage with Live/Dead staining

The cell membrane damage of the bacterial cells was determined with fluorescence microscope. 1mLsample of bacterial Fe/ZnONP slurry during PCD treatment was collected, centrifuged and washed with normal saline solution followed by staining with dyes of LIVE/DEAD® BacLight^TM^ (Life Technologies Inc., USA) bacterial viability kit according to the manufacturer’s protocol^47, 49^. The experiment was carried out using mixture of SYTO 9 dye and propidium iodide (PI) dye. Bacterial cells with intact cell membrane (live) are stained by SYTO 9 and fluorescent green whereas PI penetrates only damaged membranes and stains the dead bacterial cells. After incubation in dark for 15 min, the stained samples were examined by a Fluorescence Microscope (Floid Cell Imaging Station, Life Technology Inc, USA) with 40x magnification.

### Electron microscopy to study membrane damage

The collected bacterial sample(s) were centrifuged and washed twice with 0.9% normal saline solution^64^. 50 µL of the pellet sample dissolved in saline solution was spread on a glass slide followed by glutaraldehyde (15%) fixation and dehydration with various concentration of ethanol. The prepared slides were observed under FESEM (Nova^TM^ NanoSEM 450).

### Stability of the catalyst

The stability of the catalyst in post reaction condition was investigated using XRD. Additionally for further confirmation the post reaction water sample was analyzed using MP-AES to detect the presence of Fe^3+^ and Zn^2+^ ions.

### Solar-FeZnO assisted PCD of real water samples

To evaluate whether the Fe/ZnO assisted solar-PCD system is applicable to natural water systems, 10 L samples of each of tap (municipal supply), river, well and pond water were collected in clean and autoclaved glass bottles and stored at 4 °C. All the natural water samples were independently filtered by using Nylon membrane filter and centrifuged at 5000 rpm to remove insoluble materials, followed by autoclaving to eliminate existing microbial load. These water samples and also de-ionized water were spiked with 1.2 × 10^7^ CFU/mL of MDR *E. coli* and similar steps of solar-PCD were carried out with 0.5 g/L of Fe/ZnO NPs.

## Electronic supplementary material

Supporting Data
