# Supplementary material for: Disinfection of Multidrug Resistant Escherichia coli by Solar-Photocatalysis using Fe-doped ZnO Nanoparticles
Source: Sci Rep. 2017 Mar 7;7:104. doi: 10.1038/s41598-017-00173-0 (PMC5427922; doi:10.1038/s41598-017-00173-0)
Supplement: Supplementary file 1 — Supporting Data [file 41598_2017_173_MOESM1_ESM.pdf]

Supporting Data

Disinfection of Multidrug Resistant *Escherichia coli* by Solar-Photocatalysis using Fe-doped ZnO Nanoparticles

Sourav Das, Sayantan Sinha, Bhaskar Das, R. Jayabalan, Mrutyunjay Suar, Amrita Mishra, Ashok J. Tamhankar, Cecilia Stålsby Lundborg, Suraj K. Tripathy

Table-1 Susceptibility of *E. Coli* strain against different Antibiotics (as per CLSI guidelines)

Antibiotic	Disc Concentration (mcg/disc) As per CLSI	Zone of Inhibition (mm)	Resistance
Rifampicin	5	8	S
Cefotaxime	30	0	R
Chloramphenicol	30	20	S
Ciprofloxacin	5	0	R
Nitrofurantonin	300	20	S
Co-Trimoxazole	25	17	S
Penicillin-G	10(U)	0	R
Nalidixic Acid	30	0	R
Ampicillin	10	0	R
Ceftriaxone	30	0	R
Ceftazidime	30	0	R
Furazolidoneb	50	15	S
Cefuroxime	30	0	R
Norfloxacin	10	0	R
Tetracycline	30	0	R
Erythromycin	15	8	S
Gentamycin	10	19	S
Amikacin	30	19	S

* Zone of inhibition = Total zone (including the disc) – Diameter of the disc (6 mm)
R: Resistant, S: Susceptible , CLSI: Clinical & Laboratory Standards Institute

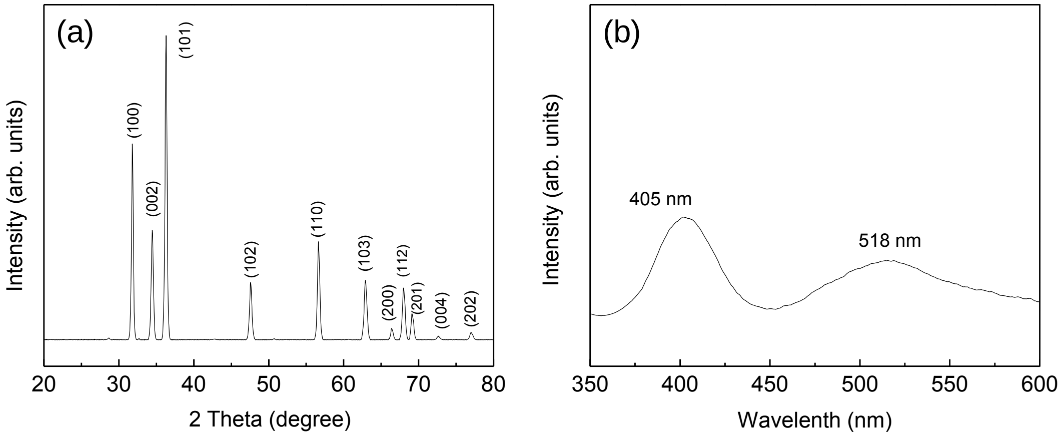

Supplementary Figure S1. (a) XRD pattern and (b) photoluminescence spectrum of ZnO NPs

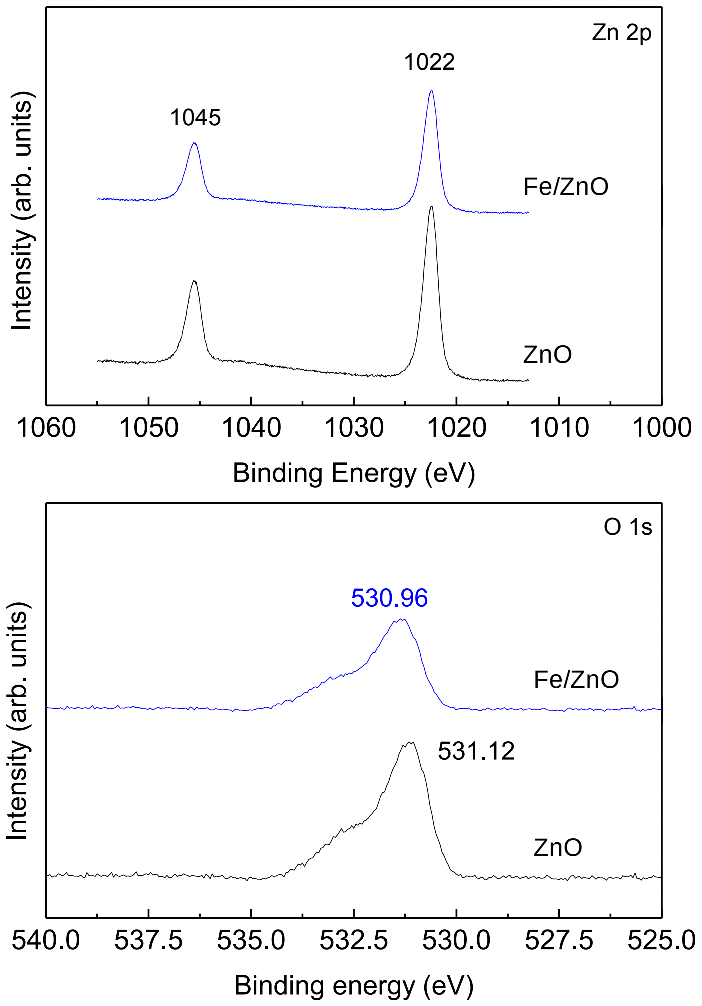

Supplementary Figure S2. XPS spectra of Zn 2p and O 1s for pure ZnO and Fe/ZnO NPs

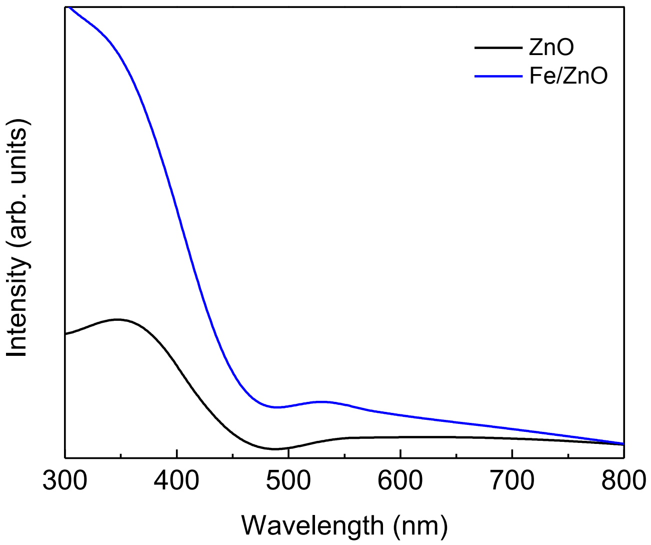

Supplementary Figure S3. UV-visible spectra of pure ZnO and Fe/ZnO NPs

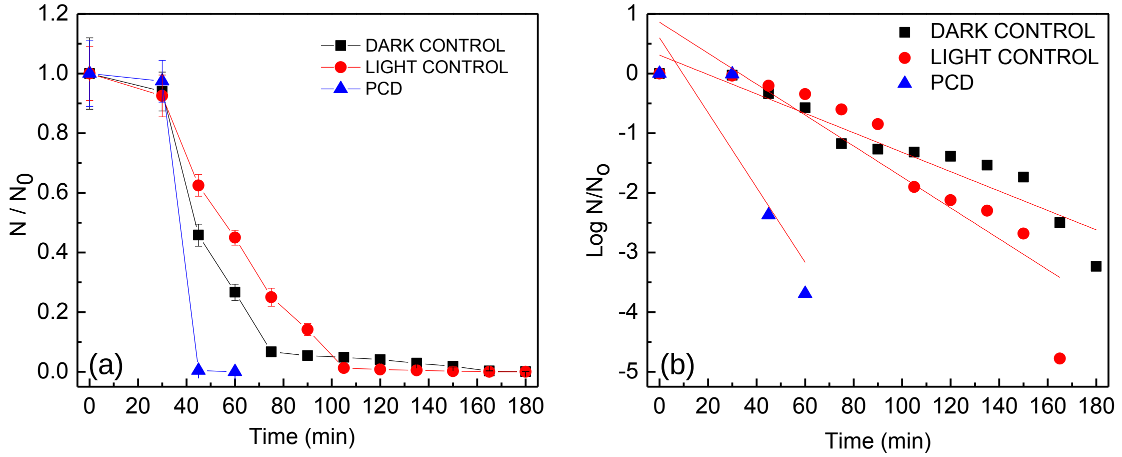

Supplementary Figure S4. (a) Effect of Fe/ZnO NPs loading on the solar-PCD kinetics of normal *E. coli*, (b) Linear fitting plots of PCD kinetics according to Chick-Watson model

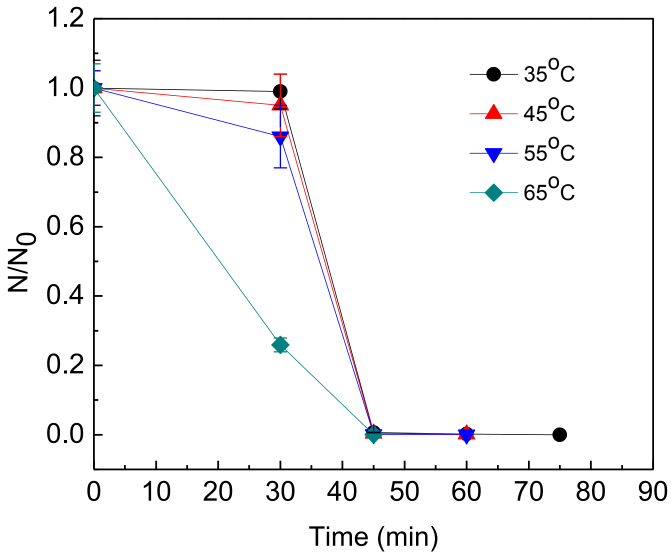

Supplementary Figure S5. Effect of reaction temperature on the solar-PCD kinetics of MDR *E. coli*. Initial MDR *E. coli* concentration = 1.2 $\times 10^7$ CFU/mL, Temperature = T±2°C (T=35^0C ~ 65^0C), pH = 6.5, [Fe/ZnO NPs] = 0.5 g/L. Error bars indicate the standard deviation of replicates (n=3).

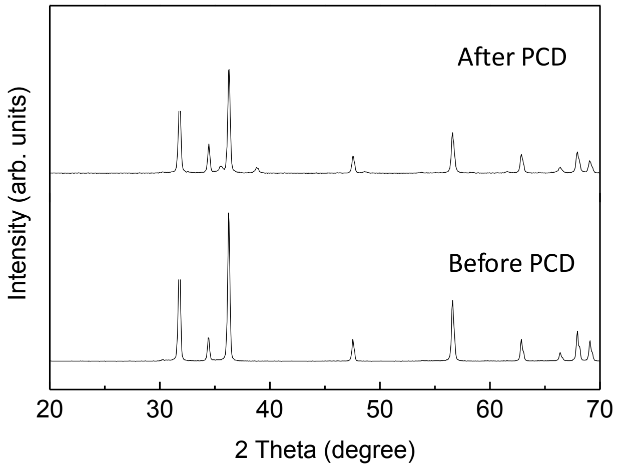

Supplementary Figure S6. XRD patterns of prepared Fe/ZnO NPs before and after recovery from the PCD experiment
